# Extending the concept of total pain to cancer survivorship

**DOI:** 10.3389/fsoc.2024.1387096

**Published:** 2025-01-30

**Authors:** Marian Krawczyk, Kari Nyheim Solbrække, Lisbeth Thoresen

**Affiliations:** ^1^End of Life Studies, School of Social and Environmental Sustainability, University of Glasgow, Glasgow, United Kingdom; ^2^Department of Interdisciplinary Health Sciences, Faculty of Medicine, University of Oslo, Oslo, Norway

**Keywords:** total pain, cancer survivorship, cancer, existential distress, suffering

## Abstract

More people are surviving cancer than ever before. While there is a growing body of research on quality of life in cancer survivorship, we still do not have a good understanding of the lived complexities that many people experience after successful treatment. Inspired by the literature on existential concerns in cancer survivorship, we consider how the concept of ‘total pain’, which emerged from the contemporary hospice movement, may be useful to think about experiences of suffering in cancer survivorship, using interviews from a Norwegian research project *Rethinking Cancer Survivorship*. We find that the concept of total pain encapsulates concerns for existential suffering and also has unique features which offer new forms of understanding and action. This includes its origins within cancer care; how it addresses the individual as a whole and re-centres the body; its reliance on and recognition of the limits of narrative; how it attends to relationality; and how the concept may afford unique insights for service development. Dying from cancer and surviving cancer are different processes, but total pain can serve as a useful conceptual compass to orient our understandings of those who experience this illness, regardless of disease outcome.

## Introduction

Cancer is one of the leading causes of death globally, and rates are increasing worldwide. In Europe alone there are more than three million new cases annually, and almost one in three people will develop cancer in their lifetime (Lagergren et al., 2019). At the same time, there have been significant advances in early detection and improved treatments, and cancer survival rates have increased substantially. As a result, almost half of these people will survive for 10 years or more, and overall survivorship is increasing by approximately 3 % annually (*Ibid*).

While there is a growing body of literature on quality of life in cancer survivorship, we still do not yet have a good understanding of the transitional experiences of cancer survivors post treatment, and there continues to be a lack of awareness about the potential late and/or long-term effects of cancer and its treatment (Ellingson and Borofka, 2018; Ueland et al., 2021a). Inspired by the literature on existential concerns in cancer survivorship, we consider how the concept of ‘total pain’, which emerged from the contemporary hospice movement, may be useful to think about experiences of suffering in cancer survivorship, using interviews from the Norwegian research project *Rethinking Cancer Survivorship*.

## Background

### Total pain

The concept of total pain was first described in the 1964 publication *Care of patients suffering from terminal illness at St Joseph’s Hospice*, by Cicely Saunders. Saunders, previously a nurse and then social worker, qualified as a doctor in 1957 and began working as a research fellow in two homes for the dying, which shaped her interest in complex pain. For Saunders, the purpose of the total pain concept was to raise awareness that there were other forms of pain (than purely physical) shaping people’s end-of-life experiences, including emotional, social, and spiritual components. These are commonly entangled, creating ‘a whole overwhelming experience’ for patients (Wood, 2022, p. 411), or in the words of one patient she often referenced, ‘it seems that all of me is wrong’ (*Ibid*: viii). Crucially, the concept of total pain developed from Saunders’ interest in patients’ social, emotional, and spiritual lives, and emerged through her – sometimes brief, sometimes in-depth – conversations with patients. It was also shaped through her deep religious convictions, and a wide range of literary sources, including existential philosophers (Clark, 2018).

By listening to and sharing patients’ words and stories, Saunders believed that the individuality and subjectivity of each person could offer insights into the end of life, while also emphasizing each patient’s unique situation, circumstances, hopes, worries, and experiences of distress and suffering. In turn, the need to address total pain became a central responsibility of the emerging field of end-of-life care and remains a foundational ideal of contemporary palliative and hospice care. For example, the World Health Organization defines palliative care as a practice ‘to improve the quality of life of patients and their families whether physical, psychological, social, or spiritual’ (World Health Organization, 2023: np). In Norway the components of total pain – and the need for holistic care to address them – are also referenced in policy documents such as the *Norwegian Official Reports* (NOU, 2017, p. 16) and the first *Norwegian White Paper on Palliative Care* (St. Meld. 24 (2019–2020)).

Saunders’ ideas were heavily influenced by her work with terminally ill cancer patients, and therefore there is unsurprisingly a significant body of literature exploring total pain in cancer patients (e.g., Ahmedzai and Boland, 2007; Brant, 2017; Mehta and Chan, 2008; Middleton-Green, 2008; Shute, 2013; Strang, 1997). The concept, however, has not yet been applied to cancer survivorship. We believe that extending the concept to people who have successfully finished cancer treatment may have significant benefits to understanding common embodied experiences in cancer survivorship, particularly in the first few years, and which we now detail further.

### Cancer and cancer survivorship

When Saunders first introduced the concept of total pain, cancer was one of the most feared diseases of the 20th century due to its ‘insidious onset and potentially devastating outcomes’ (Robb et al., 2014). At that time medical oncology included surgery, chemo- and radiotherapy; more recently, hormonal treatment, targeted therapy, and immunotherapy have been added. Many of these new treatments have remarkable success, which has led (along with advances in early detection and epidemiological and lifestyle changes) to an overall increase in cancer survivorship, even as the total number of cancer deaths continues to increase due to a largely ageing global population (Lagergren et al., 2019).

In Norway, while age-standardised rates of cancer have remained stable over the last 5 years, the number of cancer cases overall has increased by 20% in the past decade, with the most common forms of cancer being of the prostate, female breast, lung, and colon (Cancer Registry of Norway, 2022). With nearly a quarter of inhabitants aged 60 years or older, this rate is likely to increase. At the same time, nearly three out of four people in Norway survive a cancer diagnosis for 5 years or longer, and for some cancers, nearly everyone survives (Cancer Registry of Norway, 2022).

To be diagnosed with cancer in Norway is to be met with prompt response from health-care services with immediate referral to a cancer patient pathway. These are standard national pathways intended to help patients receive effective, consistent, coordinated, and predictable care (Nilssen et al., 2020). The Norwegian Cancer Society is a powerful third sector organization that also supports an individual’s needs through treatment, as well as advocacy and development of health-care policy for cancer research and treatment options (Norwegian Cancer Society, 2024). Thus, as a cancer patient, one may feel part of a wider ‘community’ of supports along with people who are going through similar experiences. After treatment is complete, depending on the type of cancer and age of the person, there are usually follow-up appointments for the next 3–5 years.

Cancer survivorship is a contested term, with varying lengths of post-treatment parameters (commonly 5–10 years), and some who live through cancer do not identify with the designation (Broom et al., 2019; Surbone et al., 2013). In part this is due to its ‘hero-vanquishing-the-adversary’ narrative and expected norms of a happy ending. As noted by Little et al. (2000) however, ‘[w]e should realize that survival is not simply the end of a story. It is the beginning of another, often very troubled, story’ (p. 502). This concern with what survivorship entails is not new. Almost 40 years ago, in the words of one physician writing about his own experiences of having survived cancer, ‘[i]t was if we have invented sophisticated techniques to save people from drowning, but once they have been pulled from the water, we leave them on the dock to cough and splutter on their own in the belief that we have done all we can’ (Mullan, 1985, p. 273). In this paper, we use the term ‘cancer survivor(ship)’ to acknowledge and bring visibility to the concept, while remaining mindful of the concerns it raises. We also reflect on these complexities as we explore the often intricate lived experiences of individuals after cancer.

Some forms of cancer are increasingly viewed as a chronic illness, with people often living for many years or decades after their initial diagnosis. People who have been successfully treated for cancer also identify numerous – often serious and debilitating – side effects from both their cancer and cancer therapies. One study found more than 25,000 publications on PubMed about the acute side effects of all cancer treatments, both during and after treatment (Lagergren et al., 2019). Many of these side effects can have long-term impact on physical, emotional, social, and spiritual wellbeing.

For example, cancer-related fatigue is one of the most common complaints of cancer patients. Fatigue often becomes chronic, extending for years into the cancer survivorship period, along with feelings of exhaustion and lack of concentration (Fitch et al., 2019; Lagergren et al., 2019; Ueland et al., 2021a). Daily functional limitations for cancer survivors may be as high as 70%, which is twice the rate for those never diagnosed with cancer (Patel et al., 2023). Other common issues include chronic pain along with diabetes, kidney and liver failure, neuropathy, lymphedema, autoimmune disorders, and infertility; as well as other physical and cognitive impairments and disabilities (Ellingson and Borofka, 2018; Hardy et al., 2018; Shapiro et al., 2018). With new therapies also come new symptoms and side effects that can cause significant health and functional impact, such as heart failure and musculoskeletal dysfunction (Lagergren et al., 2019).

Physiological and affective concerns commonly co-present. For example, one large-scale Canadian study of more than 13,000 cancer survivors (1–3 years post-treatment, various cancers) found that 87% were experiencing at least one physical concern (most commonly fatigue, change in sexual function, change in memory/concentration, and nerve problems), 78% at least one emotional concern (most commonly anxiety about cancer recurrence, depression, sadness, and loss of interest in daily activities, and decreased sexual intimacy), and 44% experienced at least one practical concern in relation to post-cancer transitioning (most commonly returning to work, getting to appointments, and health care costs), with the average number of concerns reported for each domain ranging from 2 to 3.8 (Fitch et al., 2019). While they did not discuss any connection between the domains, the study authors found that many respondents were not receiving the kind of help that would ease their transition and recovery after cancer. These issues are reflected in many other studies, which have found that between 30 and 50% of cancer survivors experience emotional and physical distress significant enough to warrant professional intervention sometime during the survivorship period, including but not limited to depression, anxiety, and sleep problems, and even post-traumatic stress disorder (Ellingson and Borofka, 2018; Knox, 2018; Swartzman et al., 2017).

### Cancer survivorship as total pain

It is important not to collapse all issues that people who have successfully been treated for cancer may face as suffering. Many cancer survivors, including those with long-term symptoms report a high quality of life and a sense of personal resilience (Costanzo et al., 2009; Ellingson and Borofka, 2018; Fitch et al., 2019). At the same time, ‘[c]ontrary to common assumptions, surviving cancer does not automatically eliminate physical, psychological, or existential suffering’ (Knox, 2020, p. 62). As part of this awareness, there is a growing body of literature on the lived experience in cancer survivorship, often focused on the transitional period; a period usually defined by survival estimates of different types of cancer. The findings of these studies highlight that during this period of post-cancer survivorship issues are not necessarily divisible into physical, emotional, social, or spiritual concerns but rather encompass the entirety of a person’s lived experience including sense of self, personal relationships and social roles, and meaning-making capacities (Ellingson and Borofka, 2018; Little et al., 2000; Surbone et al., 2013; Ueland et al., 2020; Ueland et al., 2021a). Within this research many cancer survivors have not yet transitioned into a new identity, yet also feel ‘significantly different’ from their pre-cancer selves, often accompanied by existential ambivalences from awareness of diminished capacities, loss of opportunities, and threat to one’s overall existence (Ueland et al., 2021a). Most cancer survivors wish to return to a similar life they had before being affected by cancer, even if this is no longer possible. As a cancer survivor, bodily capacity can no longer be taken-for-granted and trustworthy, and survivors describe fundamental alterations to sense of self. Relationships may alter dramatically, or alternatively, family and friends may resume pre-cancer ways of being that enhance cancer survivors’ feelings of isolation and loneliness. The future is uncertain, where cancer survivors are required to face a new reality shaped by the ambivalence of being ‘cured’ yet also at risk of further recurrence and/or heightened health challenges. In short, the integrity of the ‘whole self’ is fundamentally threatened as a result of a fundamental disruption of the person’s previous lifeworld (Ueland et al., 2020; Vehling and Philipp, 2018).

These studies illuminate how living through successful cancer treatment requires the person to face a new reality of living which may include being situated within a liminal space of uncertainty, attempting to find a new normal in everyday life, while experiencing difficulties in family functioning and communication, concerns for returning to work or school, and fear of cancer recurrence, as well as the loss of previous identity and sense of self, changed relational intimacies, and capacity to make meaning (Broom et al., 2019; Ellingson and Borofka, 2018; Knox, 2020; Ueland et al., 2020; Ueland et al., 2021a). Consequently, the resultant combination of side effects and late term effects of cancer treatment can both create and further physical, cognitive, emotional, relational, sexual, financial, social and/or existential distress, resulting in a whole overwhelming experience of suffering. These experiences can therefore result in a paradigm *sheer* rather than a paradigm *shift*, where cancer and treatment experiences disrupt continuity of self, relationships, and time in radical and fundamental ways. In the extreme, this can result in some cancer survivors questioning if the life they are left worth is really worth living (Knox, 2020).

Collectively, this literature highlights that the lived experiences of cancer survivors: (1) commonly includes a liminal (transitional) period, which (2) requires attending to both their individual embodied experiences and their social relations, and (3) can result in multifaceted suffering. These are the same concerns which gave rise to the concept of total pain. While total pain has traditionally been limited to the entanglement of physical, emotional, social and/or spiritual concerns specific to the end of life, we believe there is utility in applying the concept to cancer survivorship. Cancer survivorship is not the death of the person, yet it does engender a form of death of the old self and ending of known ways of being. Additionally, as total pain encompasses the relational aspects of suffering rather than locating it solely within the individual, the concept is useful to understand how cancer survivors are necessarily embedded within webs of relationships and collectivities, including medical practices and social norms. Finally, we believe the concept of total pain is a useful concept to interpret experiences of cancer survivorship as it focuses on both the importance and limitations of articulating embodied experiences. Some suffering resists being fully communicable. Within a total pain framing, communicating experiences of suffering can take many shapes, from lengthy and coherent narratives to evocative fragments that may point to that which is silent, wordless, or nonverbal (Wood, 2022).

## Methods

The steady number of cancer survivors is the central point of departure for the Norwegian research project *Rethinking cancer survivorship* and it is from this project that we draw interview examples that constitute our findings and the basis for our discussion (Rethinking Cancer Survivorship, 2024). The project’s main objective was to critically inquire into the category of cancer survivorship and its cultural premises and ramifications today. As part of this, one of the aims of this project is to foreground lay people’s own voices and experiences of cancer survivorship. The overall ambition of the project is to develop novel knowledge on the individual and social aspects of cancer and cancer survivorship (Solbrække and Bondevik, 2024).

As part of the sub-project *Voicing the unspeakable diseases*, interviews were conducted with 22 cancer survivors: 10 with colorectal cancer and 12 with gynecological cancers. Further demographic details can be found in Table 1. The aim of the interviews was to explore individual cancer experiences, especially what we consider as the less vocalised aspects of living beyond cancer that are socially and culturally muted. The participants were recruited through collaboration with a large urban hospital and through a national patient interest organization. Inclusion criteria included participants that had been diagnosed with colorectal or gynecological cancer; had been cancer free for the last 3–5 years; and were 18 years or older. Participant age ranged from 20 to 70 years old. The interviews followed a socio-narratology framework (Frank, 2010), and the interviews with an open invitation ‘to tell the story of your illness and life thereafter’. In addition an interview guide was used to ensure coverage of a broad range of issues including social support, pain management, intimacy and sexuality, long-term side effects, altered meaning of life and health, and altered self-identity.

**Table 1:** Participant demographics.

**Cancer type**	**Age**	**Education**	**Income**
Gynaecological	<70	College/ University	> € 55 000
Gynaecological	<70	High school	> € 55 000
Gynaecological	50-55	High school	< € 55 000
Gynaecological	50-55	High school	< € 55 000
Gynaecological	45-50	College/ University	< € 55 000
Gynaecological	>40	High school	< € 55 000
Gynaecological	>40	College/ University	> € 55 000
Gynaecological	45-50	College/ University	> € 55 000
Gynaecological	>40	College/ University	> € 55 000
Gynaecological	>40	College/ University	> € 55 000
Gynaecological	50-55	College/ University	< € 55 000
Gynaecological	65-70	College/ University	> € 55 000
Colorectal	<70	College/University	> € 55 000
Colorectal	<70	High school	< € 55 000
Colorectal	<70	High school	> € 55 000
Colorectal	65-70	College/University	> € 55 000
Colorectal	<70	High school	> € 55 000
Colorectal	60-65	College/ University	> € 55 000
Colorectal	65-70	College/University	> € 55 000
Colorectal	55-60	Elementary school	> € 55 000
Colorectal	40-45	College/ University	> € 55 000
Colorectal	55-60	College/ University	< € 55 000

All interviews were recorded and transcribed verbatim and ranged from 90 min to 3 h. Participants provided informed consent, and pseudonyms are used here. Interviews were conducted by a researcher (supervised by KS) in participants’ private homes, a cancer centre, or in a university office. We have elsewhere described and discussed these interviews as social communication processes shaped by interview location (Bjørvik et al., 2023), however all participants reflected on a range of challenges associated with cancer survivorship, regardless of interview location. Similar to many studies exploring cancer survivors’ experiences, we did not differentiate between cancer diagnoses in our analysis (Ellingson and Borofka, 2018; Fitch et al., 2019; Knox, 2020; Ueland et al., 2021a). In part this was due to the number of participants; more importantly, both forms of cancer face additional cultural stigmas and taboos in contrast to other forms of cancer as they traffic in reproductive, sexual, gender, and faecal matters. We were aware from earlier reading and analyses of the interviews that, for most of the participants, being diagnosed and going through treatment had been hard for them, and at the time of the interviews life was changed, and for many of the participants, in deeply painful ways. In the present study we make use of this pre-existing interview data to explore the benefits of bringing the concept of total pain into the field of cancer survivorship. Quotes used in this paper were collaboratively translated into English by SK and LT who are fluent and teach in both languages at a university level, as well as having researched and published extensively in both Norwegian and English.

Qualitative analysis requires analytic transparency, and we place our analysis within an initial exploratory, theory-testing framework. For the purpose of this paper, analysis employed a deductive ‘theoretically informed reading’ (Kvale and Brinkmann, 2014) of the interview transcripts, where KS and LT focused on aspects of participants’ experiences that could be interpretable first through a lens of suffering, and then extended further to total pain. They chose not to develop a systematic codebook, but rather reviewed the transcripts for descriptions or ‘evocative fragments’ of cancer survivorship which appeared to reflect a significant negative affect to the participant’s sense of self, their relationships, and/or the world around them. When these were identified, they were then translated into English and shared with the first author. All three authors collectively coded them guided by analytic findings from recent studies on the existential suffering of cancer survivors, which can be loosely grouped into three overlapping categories: (1) radical changes to embodied sense of self, (2) feelings of isolation and/or loneliness, and (3) ontological disruptions in time (Breistig et al., 2024; Knox, 2020; Ueland et al., 2020, 2021a, 2021b; Vehling and Philipp, 2018). Our goal was neither to comprehensively re-analyse every aspect of the interviews, nor to replicate or build a taxonomy of themes, but rather to consider the usefulness the concept of total pain may have in understanding the negative affective experiences of life post-cancer within existing categories of existential suffering in cancer survivorship and based on the participants’ own words. Through this categorisation we were able to consider the utility of the concept of total pain to extend existing knowledge about suffering in cancer survivorship. Our analytic strategy is therefore best considered a pilot ‘proof of application’ in that re-reading the interview transcripts was done in order to explore if total pain was a useful interpretive lens *generally* to understand key experiences of cancer survivorship and, if so, to discuss how it could become part of the conceptual toolkit for others interested in this field of study, including those focused on existential suffering. Casula et al. (2021) describe this type of analysis as deductive exploratory research; an approach which uses a ‘sensitizing concept’ to frame an ‘exploratory research process’ (p. 1704).

## Findings

Participants’ narratives were often lengthy, encompassing becoming ill, being diagnosed, going through treatment, and years of follow-up tests and appointments, and then at some point being left on one’s own. In context of their post-cancer journey, participants detailed numerous physical issues, relational concerns, and various emotions, including fear of future relapses and going through further treatments. They described how their post-cancer lives were impacted as a whole, highlighting the difficulties they faced in trying to resume or rebuild a sense of ‘normalcy’ as before, along with the struggle to establish a cohesive sense of self in their new post-cancer reality.

### Radical changes to sense of self

Previous studies have identified the ways in which cancer survivors may experience radical changes to the sense of self, in part due to new - and sometimes significant - limitations in daily life (Ellingson and Borofka, 2018; Hvidt, 2017; Knox, 2018; Little et al., 2000). We found this to be the same in our study. For example, one participant, Anders, talked about how after treatment for colon cancer ‘I suffer nearly every day’ from stomach pains and cramps, as well as with sleep problems and reduced memory. He reports that his life is now ‘directed by the stomach and the stoma’. Synne, another participant who had been told that she was cancer free, said the news left her feeling ‘completely drained and empty; life is flat’ where ‘moments of joy seldom occur’. While she wanted to do something other than work, she said ‘I disappoint myself. Why do I not do something? …I should have used more of my time and energy to build a life…I spend all my energy at work and when I get home, I just feel drained. I cannot manage’. This was in contrast to her description of herself pre-cancer, as ‘full of energy and living an active life’. Peter, a participant who required a colostomy, when talking about intimacy with his wife stated ‘We are finished with that. Life has changed’. For another participant, Ingrid, she noted the change to her sense of self quite clearly, stating that while her time after treatment has been ‘very good’ it has also been ‘absolutely terrible. Because I’m not me anymore. That person is gone. Or not all of it, but very much is gone’.

While these participants’ self can be framed through a psychosocial lens of adjustment, where cancer survivors are experiencing oversurveillance, anhedonia, depression, or anxiety, these are anemic descriptors which undervalue the real changes to some cancer survivors’ embodied experiences of self and physical capacity. Concerns for the body’s experiences and capacities are not merely about the need for adequate symptom management but are also signifiers of their new limits as embodied selves. This finding is also reflected within literature on the ways in which both cancer and the biomedical treatments to cure it can lead to ‘biographical disruption’ (Hubbard and Forbat, 2012; Trusson et al., 2016), including experiences of liminality, alienation, and even abjection towards the self and the world within which the self is situated (Hvidt, 2017; Ueland et al., 2020). Given their complexity in shaping all aspects of self, these experiences of embodied post-cancer life may resist being fully articulable, as evidenced by Peter’s terse deflection of his sexual life. At the same time, his use of ‘we’ highlights how this changed sense of self is always inextricably entwined with others.

### Feelings of isolation and loneliness

Studies have also reported on the strong sense of isolation and loneliness that many cancer survivors live with, and of ‘bearing the burden alone’, where their experiences of continuing difficulties go unrecognized (Knox, 2020; Raque-Bogdan et al., 2019; Ueland et al., 2021a). In our study, many participants described a major shift from being supported by structured cancer care routines to a post-cancer reality where they felt they had to deal with the long-term effects of the disease and treatments on their own. Participants seemed unprepared for this change, and during the interviews many of them discussed facing a new sense of medical and social indifference now they were cancer ‘free’.

Participant Silje described how, during post-treatment check-ins, she was frequently met with the brusque response that she needed to learn to live with the late effects and her changed body. She recounted health-care professionals’ orientation as ‘That’s the way it is. Goodbye. See in you half a year’. She felt that if she continued to ask questions she was perceived as ‘troublesome’. Another participant Anna also highlighted this sense of health care abandonment, which she described as ‘the feeling of being dropped…and being in deep water’. She asked, ‘Is no one going to talk with me anymore?’ Participant Bente discussed her feelings of how, during follow up appointments, she has the sense that ‘no one sees me’. Yet another participant, Synne – who prepared before going into appointments in order to advocate for herself – still felt invisible. She recounted how she would bring with her a list of questions for the specialist, yet left these consultations without asking them, feeling ‘that there was no room for my issues’. Overall, what many participants shared were feelings of being deprecated by health care professionals after treatment, even as they continued living with ongoing uncertainty concerning their current well-being and future health.

This feeling also extended to family and friends, where some participants felt there was a lack of understanding from those closest to them, and even a sense of ‘silence’, leaving them to deal with these worries alone. For example, Peter recounted that not everyone approved of him talking about his colostomy, so ‘I hide it as much as I can’. Silje, similar to Synne, also spoke about her fatigue, but here the focus was on how it challenged personal connections, in that ‘I do not have energy to meet friends after work’. Yet even if she did have the energy, she felt that ‘One talks about cancer as something you die of or survive. If I start to speak about late effects, people’s response is that I should be thankful for being alive. No one understands what surviving means – very few know about the late effects’.

This sense of abandonment, isolation and feelings of invisibility from clinicians, as well as family, friends, and colleagues are based on assumptions of survivors’ well-being when the cancer treatment comes to a ‘successful’ end. Yet as we saw in the previous section, cancer survivors may be grappling with radical changes due to alterations in physical capacities and sense of self and cannot return to the way things were before. Participants’ bodies and identities had changed through the cancer and treatment, yet these changes were not being confirmed through their relationships. As Ueland et al. (2021a)’s study of cancer survivors found, ‘*It is a double suffering*. They are a different person from the one they were, whereas in encounters with others, they are seen as they were, which enhances the distance they feel between themselves and others’ (p. 6, emphasis ours). This was also reflected in our study as many participants also described becoming more introverted and passive, spending more time on their own, and even shielding themselves from other people.

### Disruptions in time

Cancer survivors also have alterations in their relationship to past, present, and future, facing uncertainty about future health and abilities in contrast with the taken-for-granted health of the past (Tindle et al., 2019; Ueland et al., 2020). A sense of temporal continuity is one of the taken-for-granted conditions of everyday life, and both cancer and survivorship disrupt this, dividing time into a before and after (Rasmussen and Elverdam, 2007). Cancer’s return remains a constant worry, with awareness of mortality ever-present for many in the early years of cancer survivorship, and living involves facing an uncertain future while longing for a past that is out of reach, all while trying to appreciate the present (Berry-Stoelzle et al., 2020; Simonelli et al., 2017).

Participant Anders recounted this disruption where his cancer, its treatment, and fear of its return combined to create a different temporal rhythm of everyday life, where the aftermath of his surgery and the time waiting between his regular check-ups created ‘a vacuum until the next results come…at varying intervals’. Another participant, Jacob, struggled in trying not to think about his future, even as he thought about it ‘a lot’, where ‘the future I had envisioned will most likely not be like that’. For Jacob, the future is shaped by his current state, where if the cancer returns, he will not seek treatment ‘because it’s so tiring to be the way I am now’. Other participants articulated strong divisions between past, present, and future, such as Ella, who when facing concerns about the possible return of cancer stated, ‘Why take the sorrows in advance?’ and recounted turning down an offer to join a gynecology support group because ‘I do not want to be reminded’. Synne, on the other hand, evidenced absence of thinking about the past, present, or future at all, stating that what she wanted to do most of all was nothing; stating that ‘nothing is the best thing’.

Literature that considers the changed relationship with time in cancer survivorship commonly uses the anthropological concept of liminality (Hansen et al., 2019), where cancer survivors are required to navigate a threshold or boundary between ‘before’ and ‘after’; an ‘in-between’ that marks one state from another, as well as the experiences of *being within* the in-between. This temporal work of (re)structuring self and place within the world (including social relations) is rarely linear. Surbone et al. (2013) provides a particularly compelling example from a quote in the New York Times from a writer who had been diagnosed and successfully treated for cancer: ‘I’m not a cancer survivor, and neither are the women in my cancer support group…Perhaps we need a word for *that murky in-between zone* that a number of us inhabit daily’ (2,469, emphasis ours). This is similar to Ueland et al. (2021b)’s findings with cancer survivors where these types of intangible temporal feelings were part of ‘something different, which did not lend itself to classification’ (p. 706).

## Discussion

In our exploration of cancer survivors’ experiential accounts, we considered general themes already evident in the literature on existential suffering in cancer survivors and we found similarities in relation to (1) radical changes to embodied sense of self, (2) feelings of isolation and/or loneliness, and (3) ontological disruptions in time. What then are the benefits of adding a new concept such as total pain to the field? In this section we detail the following key reasons we believe it is a useful concept to discuss negative affective experiences in cancer survivorship: its origins lie largely within cancer care; it encompasses the individual as a whole and re-centres the body; both relies on and recognizes the limitations of narrative; pays attention to relationality; and finally, the concept may afford unique insights for service development.

The origins of total pain were fundamentally shaped by Saunders’ work with end-of-life cancer patients (Clark, 1999). Total pain is therefore a particularly relevant lens to understanding experience of suffering in cancer survivorship because it directly emerges from, and engages with, peoples’ concerns with mortality in relation to other forms of pain specifically within experiences of cancer. This is a significant point as cancer diagnosis, treatment, and survivorship ‘reverberates in people’s lives in a different, deeper and more pervasive way than a diagnosis of heart failure or other diseases’ (Surbone et al., 2013, p. 2470), with perhaps the exception of dementia. Extending the concept of total pain to experiences of cancer survivorship may therefore enable a particular sensitisation to issues that are cancer specific – such as concerns for recurrence, noted by several participants.

The idea of existential distress or suffering within health care literature is often not well defined (Boston et al., 2011; Strang et al., 2004). From one perspective, the same claim can also be levied against total pain. In his investigation of the textual heritage of the concept since it was first used in the early 1960s, Joe Wood highlights how Saunders’ herself never provided a singular definition of total pain, and he traces how it has changed meaning in different times and contexts (2021, see also Krawczyk and Richards, 2018). At the same time, the concept of total pain is explicitly built on the need to give equal attention to physical, social, emotional, and existential issues *as well as* how they interact to become more than the sum of their parts. While these components are divided into ‘constituent parts’ within clinical and palliative care literature, ‘in later life Saunders herself was keen to note that any separation of total pain into physical, mental, social, spiritual…pain represents an artificial division of ‘a whole overwhelming experience” (Krawczyk et al., 2018, p. 3). Perhaps the most interesting aspects of the interviews with the cancer survivors discussed here is how they at times narrated their lives post-cancer in ways that ‘slip between categories’; and therefore, not easily addressed or cured within any one area of consideration (Knox, 2020). As Silje recounted ‘One talks about cancer as something you die of or survive…no one understands what surviving means’. The concept of total pain maps out different arenas of pain while simultaneously advocating their indivisibility in some peoples’ experiences of cancer survivorship after successful treatment.

This leads to an overlapping aspect; how total pain foregrounds the centrality of the body as the necessary ground of all experience. ‘Existential suffering’ is an important concept in cancer survivorship, yet the term may often be reduced to abstract perceptions of mind, self, and meaning making. As a result, the ‘fleshy’ aspects of the experiencing body may be unintentionally backgrounded. As Anders noted, he is ‘directed by the stomach and stoma’, and Peter hides as much of his changed body as he can. The use of the word ‘pain’ within total pain draws us to an understanding that it is the complex biopsychosocial interaction between bodies and environments (cultural, material, biological) which fundamentally shapes our capacities to be in the world.

Total pain has been historicised as reflecting and creating a holistic understanding of the dying patient’s situation within 20th century medicine. Rather than a purely physical aetiology, the meaning of a dying patient’s pain and its resolution was made possible only by attending to their narratives as they came close to the ending of their own story. Wood (2021) considers ‘Perhaps ‘total pain’ felt like a narrative term because it articulated the pain of being ‘total’, of being finite and coming up against that finitude’ (p. 91). Similar to late-stage cancer patients nearing the end of life, the effects of cancer survivorship can be hard to observe or trace, where experiences may resist cohesive, linear, and well-developed narratives, such as Peter’s terse response that ‘we are finished with that [having a sexual relationship]’ (see also Knox, 2018; Surbone et al., 2013; Ueland et al., 2021a). This may be one reason why Saunders often illustrated examples of total pain using short anecdotes and evocative fragments rather than lengthy narratives, as an epideictic effect to ‘invite, rather than offer, narrative interpretation’ (Wood, 2022, p. 5) and to highlight the importance of ‘documenting the language and metaphors which her patients use[d]’ (*Ibid*: 16) to describe their experiences. We see these metaphors in Synne’s expression that ‘life is flat’, and Anna’s feeling ‘of being dropped… in deep water’.

This close attention to expressions of embodied experiences is closely entwined with our next point; that total pain requires an understanding that suffering has relational elements, and that the antecedents of suffering (as well as its effects and amelioration) often extends well beyond the individual. People are embedded in webs of relationships, and employing the concept of total pain in cancer survivorship requires us to attend to relations that extend far beyond family and friends, including health care professionals^1^. This introduces our final point; how the concept of total pain identifies the central importance of health care relationships. In order to attend to patients’ embodied experiences, Saunders advocated a reframing of the relationship between health care providers and patients, where the ‘question should not be what do you tell your patients but what do you let your patients tell you?’ (Wood, 2022, p. 7). This way of attending asks that health care professionals ‘decolonis[e] the patient experience from biomedicine and acknolwedg[e] the expertise of the patient’ (Gunaratnam, 2012, p. 15). This also requires the ability to engage with emotional complexity by attending to what the patients says through carefully listening and striving to be present even (especially) when articulation may be difficult or impossible. For Saunders, witnessing – being with – suffering was an imperative even it could not be resolved as the end of life neared. She believed that ‘never let it be said nothing more can be done’, as one of the most powerful acts another person could do was to sit and be in witness together – in her words, to ‘watch with me’ (Krawczyk et al., 2018).

Gunaratnam (2012) has suggested this way of being can also be understood through Keat’s notion of ‘negative capability, that is, when a man [sic] is capable of being in uncertainties, mysteries, [and] doubts’ (cited in: Coulehan, 2017, p. 2429). Trauma, suffering, and loneliness—sometimes these modes cannot be helped; yet the person must live with them. In such circumstances, health professionals can sit with people in their suffering while ‘tolerating ambiguity and not-knowing’ (Gunaratnam, 2012, p. 23). We suggest this approach to considering health care providers’ capability also highlights there is always more to human life than what can be measured; it is also defined by what we do not know and/or cannot control. Not everything can be fully explained or verbalized, either to oneself or others; yet even in these ineffable moments one can still be present. We suggest that being with the complexities of experiences identified here requires negative capability, and in turn engenders further capability by so doing. This is a hard ask for health care professionals for a number of reasons, including that it differs from biomedical perspectives where focus primarily centres on the development and use of standardised guidelines and screening tools to reduce survivors’ unmet needs, for example by using a ‘distress thermometer’ (e.g., Emery et al., 2022). However, to listen and acknowledge others’ experiences may sometimes be enough; ‘to listen actively and generously to the winding, stony road to selfhood post cancer’ (Knox, 2020, p. 70).

We also offer two additional practical points regarding the benefits of the concept of total pain in assisting health care providers working with cancer survivors: the requirement of multidisciplinary involvement, and better institutional intelligibility. Saunders’ asserted that a multidisciplinary approach was needed to engage with total pain. This approach has become known as ‘total care’, ‘whole person care’, and ‘holistic care’, all which are now terms commonly used in health care. Employing the concept of total pain in cancer survivorship alerts us to the benefits of a holistic engagement which requires involvement beyond clinical management of symptoms. As noted by MacDonald et al. (2021) ‘By embracing principles of palliative care, survivorship care may be guided by a theoretical foundation that provides cancer survivors with care that supports increased [quality of life], biopsychosocial symptom management, and a holistic perspective of the illness experience’ (p. np). In addition, the concept of total pain may have better institutional intelligibility within health care rather than the more amorphous concept of suffering, as the former term was developed within medical settings, and the latter term potentially places these kinds of experiences as outside the realm of health care. Total pain also echoes our own figurative use of the word ‘pain’ in everyday language (Krawczyk et al., 2018). As noted by Bueño-Gomez (2017) in his work on conceptualising pain and suffering, ‘[d]efinitions are not inconsequential, since the way in which we define concepts has epistemological, ontological and practical dimensions’ (p. 1). While we do not advocate the reach or responsibility of health services into every aspect of human experience, as noted earlier, cancer survivors actively look for health care professionals’ awareness and understanding of their post-cancer experiences. Attending to the early journey after cancer treatment is a critical time for many as biomedical treatments may have ‘cured’ the cancer but can also leave people feeling alienated from their lived bodies and relationships, and where their experiences of the world are no longer the same.

We also do not want to constitute post-cancer experiences as uniform, or uniformly negative. This was clear in Ingrid’s expressed sense of her post-cancer self as both ‘very good’ and ‘absolutely terrible’. At the same time, it is clear that many people who have undergone successful treatments for cancer also go through transitional and transformational processes that are influenced by many individual, inter-, and intra- personal factors as they engage in ongoing inner work of trying to find a way to make sense of new limitations or changes in identity and everyday life. This period appears to commonly include a liminal zone where embodied experiences may be unsettled, shifting, and context dependent as individuals navigate the borderless and unfamiliar territory of becoming a cancer survivor (Hvidt, 2017; Ueland et al., 2020). Consequently, the concept of total pain may be particularly relevant during the period where individuals have become unmoored from their lives pre-cancer, facing the ending of the previous self and changing relationships, and striving to endure through their liminal transition, before moving fully into and integrating new experiential terrain. Ueland et al. (2021b) frames this journey as the striving to bring together two lifeworlds – the old ‘home world’ and new ‘alien world’. Rather than the notion of recovery, which implies return to a previous state, cancer survivors may be more accurately described as facing the work of self-becoming over time in ways that requires accepting the death of who they used to be, which – for some – is a painful, and poorly understood, process.

## Limitations

We are conscious of a number of limitations to our thinking, methods, and analysis. First, the concept of total pain is ‘slippery’, with diverse definitions and applications. As a consequence, it can become difficult to meaningfully apply to cancer survivorship experiences, and risks becoming little more than ‘pop holism’ (Wood, 2021). We have tried to address this by providing a clear definition of total pain as well as tracing its origin within cancer care, and included ways in which it can generate specific actions by health care providers. We are also aware that the idea of total pain can be used as a normative ideal within health care, where attending to narratives of distress and suffering requires that all aspects of the self be open to the medical gaze. This also resonates with our discomfort with the word ‘survivor’ which is differentially defined, does not resonate with many of the people it attempts to describe, and implies that having cancer is a battle with a clearly defined enemy. Our use of the term has perpetuated this issue, even as we sought to problematize it, which we have done for visibility needed in key words and search terms. Finally, we are aware of the methodological limitations of a relatively small sample size and our analytic procedures. Many cancer survivorship studies have modest numbers of participants, and our study was no exception. However, as we stated at the outset, our aim was exploratory: to consider whether the concept of total pain may have value in deepening our understanding of negative affective experiences in cancer survivorship. We leave it to others to extend this conceptual exploration in a more systematic manner. While our analytical process was somewhat unorthodox, we have tried to ensure methodological transparency.

## Conclusion

More people are surviving cancer than ever before. In tandem there is a growing body of research exploring the complex experiences of living a ‘post’ cancer life. Our aim in this article was to consider the relevance of the concept of total pain to understand significantly negative affective experiences in cancer survivorship, particularly in relation to the transitional period of 3–5 years after successful treatment. We found that total pain mapped on to exiting categories of existential suffering, and we articulated how the concept of total pain may be uniquely useful to thinking about, and engaging with, cancer survivors. Dying from cancer and surviving cancer are different processes, but total pain may serve as a useful conceptual compass for understanding the experiences of this illness, regardless of disease outcome.

## Data Availability

The data analyzed in this study is subject to the following licenses/restrictions: the interviews contain sensitive information that contain potentially identifying and sensitive information and is therefore not accessible. Requests to access these datasets should be directed to https://www.med.uio.no/helsam/english/research/projects/cancul/.

## Footnotes

^1^Intriguingly, from the early 1980s onward Saunders at times included ‘staff pain’ within her discussions of total pain to include the impact on staff of work with dying patients.
